# A retrospective study of conscious sedation versus general anaesthesia in patients scheduled for transfemoral aortic valve implantation: A single center experience

**DOI:** 10.1002/hsr2.95

**Published:** 2018-11-01

**Authors:** Jochen Renner, Anna Tesdorpf, Sandra Freitag‐Wolf, Helga Francksen, Rainer Petzina, Georg Lutter, Norbert Frey, Derk Frank

**Affiliations:** ^1^ Department of Anaesthesiology and Intensive Care Medicine University Hospital Schleswig‐Holstein Germany; ^2^ Department of Trauma Surgery University Hospital Schleswig‐Holstein Germany; ^3^ Institute of Medical Informatics and Statistics Kiel University Germany; ^4^ Department for Cardiovascular Surgery University Hospital Schleswig‐Holstein Germany; ^5^ Department of Cardiology and Angiology University Hospital Schleswig‐Holstein Germany

**Keywords:** anaesthesia techniques, aortic stenosis, TAVI, sedation

## Abstract

**Objectives:**

The current 2017 ESC/EACTS guidelines recommend transcatheter aortic valve implantations (TAVIs) as the therapy of choice for inoperable patients with severe symptomatic aortic stenosis. Most of the TAVIs worldwide are performed under general anaesthesia (GA). Although conscious sedation (CS) concepts are increasingly applied in Europe, it is still a matter of debate which concept is associated with highest amount of safety for this high‐risk patient population. The aim of this single center, before‐and‐after study was to investigate feasibility and safety of CS compared with GA with respect to peri‐procedural complications and 30‐day mortality in patients scheduled for transfemoral TAVI (TF‐TAVI).

**Methods:**

From March 2012 until September 2014, patients scheduled for the TF‐TAVI procedure were included in a prospective, observational manner. From the 200 patients finally included, 107 procedures were performed under GA, using either an endotracheal tube or a laryngeal mask, and balanced anaesthesia. CS was performed in 93 patients using low‐dose propofol and remifentanil.

**Results:**

Conversion to GA was needed 4 times due to procedural‐related complications (4.3%), in one patient due to ongoing agitation (1.1%). The CS‐group showed significantly shorter key time courses: anaesthesia time (105 [95‐120] minutes vs 115 [105‐140] minutes, *P*‐value = 0.009, Mann‐Whitney‐U‐test) and length of stay in the intensive care unit (1.6 [1.0‐1.5] d vs 2.1 [1.0‐2.0] d, *P*‐value = 0.002, Mann‐Whitney‐U‐test). The lowest mean arterial pressure was significantly higher in the CS‐group compared with the GA‐group (74.3 mmHg vs 55.2 mmHg, *P*‐value <0.0001, t‐test). CS was associated with less requirements of norepinephrine (0.1 μg/kg vs 2.3 μg/kg, *P*‐value <0.0001, Mann‐Whitney‐U‐test).

**Conclusions:**

Our single‐center data demonstrate that CS is a feasible and safe alternative, especially with respect to a higher degree of intra‐procedural haemodynamic stability, and a reduced length of stay in the intensive care unit.

## 1 INTRODUCTION

Aortic valve stenosis is one of the most common acquired valvular heart diseases in the elderly and, generally, a major cause of morbidity and mortality.^1^, ^2^, ^3^ For many years, the only available options for patients classified to be inoperable were conservative medical treatment and/or balloon aortic valvuloplasty. However, both options must be considered as a palliative approach, since the benefit regarding improvement in outcome is highly limited.^4^ In 2002, Cribier et al reported the first experience with transfemoral transcatheter aortic valve implantation (TF‐TAVI) in patients with inoperable end‐stage aortic stenosis.^5^ To date, it is estimated that more than 200 000 TAVI procedures have been performed worldwide, and it has been established as a minimally invasive technique for this patient population.^6^ Nearly 70% to 80% of TAVI procedures have been performed using the transfemoral approach (TF‐TAVI).^7^ Increasing experience, device‐specific improvements, and economic aspects coupled with the possibility to generally perform TF‐TAVI procedure under conscious sedation (CS) have led to an ongoing debate about the anaesthesiological management associated with the highest degree of safety for this patient population.^8^, ^9^, ^10^ However, worldwide, approximately 90% of the TF‐TAVIs are operated on general anaesthesia (GA) using transoesophageal echocardiography (TOE) guidance.^11^, ^12^ Between 2008 and 2015, more than 48 000 TAVI procedures were documented in Germany, showing a considerable higher amount of TF‐TAVIs compared with the transapical and transaortic surgical approach^13^ Several investigations have demonstrated that CS is a feasible and safe concept for TF‐TAVI procedures.^10^, ^14^, ^15^ In consideration of the ongoing high proportion of procedures worldwide performed under GA, the objective of the present retrospective analysis of 200 TF‐TAVIs was to evaluate the feasibility and safety of our CS‐concept compared with GA, and to report peri‐procedural complications, main time courses, and 30‐day mortality in this patient population. Since the logEuroSCORE I is used to calculate the predicted perioperative mortality of patients undergoing cardiac surgery, we moreover hypothesized that patients presenting a logEuroSCORE *I* ≥ 22% might benefit more from a CS‐concept than from GA.

## 2 METHODS

This registry complies with the “Declaration of Helsinki,” and the retrospective study was conducted in accordance with the Guidelines for Good Clinical Practice and approved by our institutional ethical committee (Ethikkommission UKSH Kiel—AZ D529/16, Christian‐Albrechts‐University Kiel, Schwanenweg 20, D 24105 Kiel), with waiver of informed consent.

The TAVI procedure has been established in our hospital since 2008. In the first period (2008‐2011), GA was the solely used anaesthesia technique. Regarding the influence of the “learning curve” on the first TF‐TAVIs in our hospital, we decided, under consideration of the available literature, to exclude the procedures performed between 2008 and 2011.^16^, ^17^ From March 2012 until September 2014, a total of 200 consecutive cases were included in a prospective, observational manner. Initially, all procedures were performed in the cardiac catheterization laboratory, and since 2013, in a new hybrid operating room (OR). A perfusion technician was always available to set up emergent extracorporeal circulation, if necessary. With the opening of the new hybrid OR, our Heart Team decided to change the first line anaesthesia management from GA (*n* = 107) to CS (*n* = 93), which is the preferred technique in our center up to now. Patients undergoing transapical or transaortic TAVI were excluded from this analysis. In regard to our hypothesis that patients at higher risk of periprocedural mortality, defined by a logEuroScore ≥22%, might benefit more from a CS‐concept than from GA, we built subgroups of patients with a logEuroSCORE I </≥22%, which has been the calculated median value of our patient population. We, thereafter, compared the GA‐group with a logEuroSCORE *I* ≥ 22% (*n* = 52) with the CS‐group with a logEuroScore I ≥ 22% (*n* = 47) in consideration of peri‐procedural complications, main time courses, and 30‐day mortality.

### 2.1 Anaesthesia techniques

Two consultant anaesthetists with many years of comprehensive experience in the field of cardiac surgery and TAVI procedures were responsible for the anaesthesiological management. All patients were monitored with a five‐channel electrocardiogram, pulse oximetry, invasive radial artery blood pressure, and a central venous catheter. A heating blanket was positioned underneath the patient, and warmed intravenous fluids were given. In both groups, two external adhesive radio‐transparent defibrillator pads were attached to the patient for early treatment of procedure‐related episodes of ventricular fibrillation. GA, as well as CS, were provided by two qualified cardiothoracic anaesthesiologists.

### 2.2 General anaesthesia

In the GA‐group, patients received low‐dose midazolam i.v. (0.01‐0.02 mg/kg) prior to the beginning of the anaesthesia preparations. After insertion of a radial artery line under local anaesthesia (LA) for continuous blood pressure measurement, GA was induced with a bolus of propofol 1 to 1.5 mg/kg or etomidate 0.15 to 0.3 mg/kg body weight, followed by a continuous infusion of propofol (3‐5 mg/kg/h), along with continuous infusion of remifentanil (0.3‐0.4 μg/kg/min) and a bolus of rocuronium (0.5‐0.6 mg/kg). Airway management was performed using a single lumen endotracheal tube or a laryngeal mask. Subsequently, a multi‐lumen central venous catheter and a venous sheath were inserted. In the GA‐group undergoing endotracheal intubation, transoesophageal echocardiography (TOE) was performed, whereas in patients ventilated via a laryngeal mask, transthoracic echocardiography was applied. Urinary catheterization was performed, and bladder temperature measured. Whenever possible, extubation of the patients at the end of the procedure was performed. After the intervention, all patients were transferred to the intensive care unit (ICU) or to the intermediate care station (IMC), for postoperative monitoring.

### 2.3 Conscious sedation

In the CS‐group, patients received low‐dose midazolam i.v. (0.01‐0.02 mg/kg) prior to the beginning of the anaesthesia preparations. After insertion of a radial artery line and the central venous catheter under LA, propofol (0.3‐0.5 mg/kg/h) and remifentanil (0.02‐0.06 μg/kg/min) were administered in low dose to keep the patients comfortable, cooperative, and capable of controlling the airway. Supplemental oxygen by face mask (2‐6 L/min) was given during the intervention. A capnography was established in order to continuously monitor breathing activity. Additionally, 5 to 10‐mL scandicain 1% for LA was injected subcutaneously in each groin at the vascular access sites. Transthoracic echocardiography was performed prior to the start of intervention and at the end of the procedure. After the intervention, all patients were transferred to the ICU or to the IMC, for postoperative monitoring.

### 2.4 Transfemoral transcatheter aortic valve implantation

The current 2017 ESC/EACTS guidelines recommend TAVI as the therapy of choice for inoperable patients with severe symptomatic aortic stenosis. In addition, the ESC/EACTS suggest TAVI as an alternative to surgical aortic valve replacement in patients with high operative risk.^18^ The current update of the AHA/ACC guidelines even advises to consider TAVI in patients with intermediate risk.^19^ At our center, patients estimated either inoperable or at elevated risk for a conventional surgical procedure were evaluated by an institutional Heart Team, composed of cardiologists, cardiothoracic surgeons, and anaesthetists.

In general, the interventional procedures were carried out by the same two cardiologists, using mostly valves from the balloon‐expandable Edwards Sapien family according to the instructions for use (Edwards Sapien, No. 9000TFX, Sapien XT, No. 9300TFX, and Sapien‐3, No. 9600TFX heart valve system, Edwards Lifesciences, Irvine, CA, USA). A total of *n* = 4 self‐expandable CoreValve prosthesis were implanted (GA, *n* = 2; CS, *n* = 2) (MCS‐P3 prosthesis family, Medtronic, Minneapolis, MN, USA). High‐resolution fluoroscopy and contrast aortography were used as imaging methods. Prior to the balloon aortic valvuloplasty and the implantation of the balloon‐expandable prosthesis, a transvenous pacing lead was implanted into the right ventricle. A rapid ventricular pacing rate of 180 beats per minute was initiated to reduce pulsatile transaortic flow and consequently pulse pressure, in order to minimize the risk of malpositioning of the valved stent. Self‐expandable valves were implanted after predilatation using a ventricular pacing rate of 110 to 120 beats per minute to facilitate optimal valve positioning. Closure of the arterial vascular access site was achieved using the two Proglide or one Prostar XL vascular closure system(s) (both Abbott, Chicago, IL, USA).

### 2.5 Data collection

#### 2.5.1 Patients characteristics and peri‐procedural parameters

Information about demographics, co‐morbidities, NYHA and ASA classification, concomitant diseases, chronic medication, biochemical data, and laboratory parameters were obtained from the patients records with a follow‐up time of 30 days after the intervention, according to internal standards. The STS score and the logEuroSCORE I have been described in detail elsewhere.^20^, ^21^, ^22^, ^23^ Procedural data were obtained from the anaesthetic protocols, which were evaluated retrospectively. Peri‐procedural‐related parameters were defined as total amount of propofol, remifentanil, norepinephrine, epinephrine, amiodarone, maximum and minimum of the mean arterial pressure (MAP) and heart rate, total amount of administered i.v. fluids and red blood cells, platelets, and fresh frozen plasma transfused. During the time period of valve implantation, the rapid pacing maneuver, as a basic prerequisite, is purposely induced to reduce ventricular stroke volume and consequently blood pressure, independent of the type of anaesthesia performed. Consequently, this time period has been excluded from data analysis.

#### 2.5.2 Postoperative complications and outcome variables

Postoperative data included the ICU stay, the follow‐up, echocardiographic parameters, and laboratory parameters. Postoperative complication and outcome variables were defined according to Valve Academic Research Consortium^24^, ^25^ as follows: 30‐day mortality, peri‐procedural acute myocardial infarction, stroke or TIA, bleeding (major, minor, life threatening), acute kidney failure (up to 48 hours post‐procedural), vascular complications, need for permanent pacemaker, pleural effusion, and pericardial effusion. The following key time courses have been defined: total time of anaesthesia, procedural time (time from the applied LA in each groin until the final closure of the vascular access site), length of stay (LOS) in the operating room (OR), time to first mobilization, and LOS in the ICU and in the hospital.

#### 2.5.3 Statistical analysis

Statistical analyses were performed using PASW statistics 24 software (SPSS, Chicago, USA). All continuous variables were tested for normal distribution with the Kolmogorov‐Smirnov test and according to this result, summarized as mean with standard deviation or median with lower and upper quartile. For the comparison of the mean, the t‐test was performed; for not normally distributed data, the Mann‐Whitney‐U‐test was used. Discrete variables were compared using the chi‐squared‐test. All tests were two‐tailed at a significance level of *P* < 0.05. A log rank test was performed for all time‐to‐event data, after dichotomizing the continuous parameters at the median. All variables with significant log‐rank tests were used for Cox regression analysis. Based on forward selection (likelihood ratio criteria), independent risk factors were identified, and the type of anaesthesia was then added into the model. The impact of these factors upon the survival time is presented as hazard ratio (HR) with 95% confidence intervals (CI), and assessed by Wald‐test. The Kaplan‐Meyer curves were plotted using the software R (Version 3.3.2).

## 3 RESULTS

### 3.1 Baseline patients' characteristics

Our analysis included 200 patients, 107 undergoing GA and 93 undergoing CS. Patients' characteristics are shown in Table 1. The mean (SD) age of patients in the GA‐group was 82 years (6.1), while it was 82 (6.4) years in the CS‐group. Although the groups were not randomized but defined by anaesthesia technique, the groups showed only a significant difference with respect to mean height (GA: 167 [8.7] cm vs CS: 170 [9.6] cm, *P*‐value = 0.035, t‐test). No difference, however, was observed for the mean BMI (GA: 25.98 [5.3] kg/m^2^ vs CS: 26.4 [5.3] kg/m^2^, *P*‐value = 0.553, t‐test).

**Table 1:** Patient characteristics

	Overall (*n* = 200)	GA‐Group (*n* = 107)	CS‐Group (*n* = 93)	*P‐*Value
Age (yr) a	82 (6.2)	82 (6.1)	82 (6.4)	0.828
Height (cm) a	169 (9.2)	167 (8.7)	170 (9.6)	0.035
Weight (kg) a	76.2 (17.7)	74.2 (15.6)	78.4 (19.7)	0.095
BMI (kg/m^2^) a	26.2 (5.3)	25.98 (5.3)	26.4 (5.3)	0.553
Sex, male b	92 (46)	44 (41.1)	48 (51.6)	0.138
Diabetes mellitus b	55 (27.5)	34 (31.8)	21 (22.6)	0.146
Hypertension b	181 (90.5)	97 (90.7)	84 (90.3)	0.936
Dyslipidemia b	101 (50.5)	50 (46.7)	51 (54.8)	0.253
Glomerular filtration rate (mL/min) c	56 (41‐60)	56.5 (40‐60)	54 (42‐60)	0.872
Coronary artery disease b	132 (66)	66 (61.7)	66 (71)	0.167
Cerebrovascular disease b	37 (18.5)	21 (19.6)	16 (17.2)	0.660
COPD b	25 (12.5)	14 (15)	11 (11.8)	0.789
Atrial fibrillation b	86 (43)	44 (41.1)	42 (45.2)	0.565
Previous cardiac surgery b	66 (33)	38 (35.5)	28 (30.1)	0.417
Pacemaker b	31 (15.5)	17 (15.9)	14 (15.1)	0.871
NYHA I b	12 (6)	6 (5.5)	6 (6.5)	0.793
NYHA II b	57 (28.5)	28 (26.2)	29 (31.2)	0.793
NYHA III b	113 (56.5)	64 (59.8)	49 (52.7)	0.793
NYHA IV b	18 (9)	9 (8.4)	9 (9.7)	0.793
LogEuroSCORE I a	27.1 (18.1)	26.2 (18.2)	28.0 (18.1)	0.486
STS score a	6.3 (4.3)	6.2 (3.5)	6.6 (5.1)	0.630

a Mean (standard deviation; *P*‐value t‐test).

b Numbers (% of total in group; *P*‐value t‐test).

c Median (interquartile range; *P*‐value Mann‐Whitney‐U‐test).

Abbreviations: BMI, body mass index; COPD, chronic obstructive pulmonary disease; EuroSCORE, European System for Cardiac Operative Risk Evaluation; NYHA, New York Heart Association; STS score, Society of Thoracic Surgeons.

### 3.2 Conversion from CS to GA

A conversion from CS to GA due to procedural‐related complications was needed in five cases (5.1%) due to different reasons: in four cases (4.3%), conversion from CS to GA was related to procedural complications, while in the remaining patient (1.1%), this was done due to persistent agitation (Supplementary Table S1).

### 3.3 Intra‐procedural anaesthesia characteristics

The anaesthesia‐related procedural variables can be found in Table 2. The median (IQR) anaesthesia time and the procedural time in the GA‐group (median anaesthesia time: 115 [105‐140] minutes; procedural time: 55 [40‐70] minutes) were significantly longer compared with those in the CS‐group (median anaesthesia time: 105 [95‐120] min, *P*‐value = 0.009, Mann‐Whitney‐U‐test; procedural time: 45 [35‐55] min, *P*‐value = 0.019, Mann‐Whitney‐U‐test). The total amount of given anaesthetics, mainly propofol and remifentanil, were significantly higher in the GA‐group compared with the CS‐group (*P*‐value <0.0001, Mann–Whitney‐U‐test). With respect to reaching and/or maintaining haemodynamic stabilization, significantly more volume and more vasopressors like norepinephrine and Akrinor were administered in the GA‐group. Nevertheless, we observed a significantly lower value of MAP in the GA group compared with CS (mean MAP, GA: 55.2 [17.1] mmHg vs MAP, CS: 74.3 [17.1] mmHg, *P*‐value <0.0001, Mann‐Whitney‐U‐test). Additionally, transfusion rate of red blood cells in the GA‐group was significantly higher compared with the CS‐group.

**Table 2:** Intra‐procedural anaesthesia characteristics

	Overall (*n* = 200)	GA‐Group (*n* = 107)	CS‐Group (*n* = 93)	*P‐*Value
Anaesthesia time (min) a	110 (96‐130)	115 (105‐140)	105 (95‐120)	0.009
Procedural time (min) a	49 (35‐70)	55 (40‐70)	45 (35‐55)	0.019
Propofol total (mg/kg) a	1.8 (0.5‐3.6)	3.5 (2.9‐4.3)	0.5 (0.3‐0.6)	<0.0001
Remifentanil total (μg/kg) a	7.7 (1.8‐15)	14.6 (12.3‐17.2)	1.8 (1.4‐2.5)	<0.0001
Volume total (mL/kg) a	7.5 (5‐10)	8.0 (5‐11)	7.0 (5‐9)	0.047
Norepinephrine total (μg/kg) a	*n* = 183 1.4 (0.1‐3.3)	*n* = 102 2.3 (1.4‐4)	*n* = 81 0.1 (0.1‐1.2)	<0.0001
Akrinor* given *n* (%) b	30 (15)	30 (28)	0	<0.0001
Amiodarone given *n* (%) b	11 (5.5)	6 (5.6)	5 (5.4)	0.94
Enoximon given *n* (%) b	7 (3.5)	3 (2.8)	4 (4.3)	0.56
MAP max (mmHg) c	95.8 (13.4)	95.3 (14.3)	96.4 (12.4)	0.65
MAP min (mmHg) c	64.1 (17.3)	55.2 (17.1)	74.3 (10.7)	<0.0001
HR max (/min) c	74.1 (15.3)	74.1 (16.2)	74.1 (14.4)	0.99
HR min (/min) c	54.4 (16.2)	48.6 (16.6)	61.1 (13.0)	0.001
SpO_2_ min (%) a	94 (92‐96)	94 (92‐97)	93 (91‐95.5)	0.54
Red cell unit given =1 *n* (%) b	7 (3.5)	5 (4.7)	2 (2.2)	0.05
Red cell unit given >1 *n* (%) b	12 (6)	10 (9.3)	2 (2.2)	0.05
Platelets given *n* (%) b	4 (2)	4 (3.7)	0	0.06
FFP given *n* (%) b	1 (0.5)	1 (0.9)	0	0.35

a Median (interquartile range; *P*‐value Mann‐Whitney‐U‐test).

b Numbers (% of total in group; *P*‐value t‐test).

c Mean (standard deviation; *P*‐value t‐test),

Abbreviations: CS, conscious sedation; FFP, fresh frozen plasma; GA, general anaesthesia; HR, heart rate; MAP, mean arterial pressure.

### 3.4 Peri‐procedural complications, main peri‐procedural time courses, and mortality

**Figure 1:**
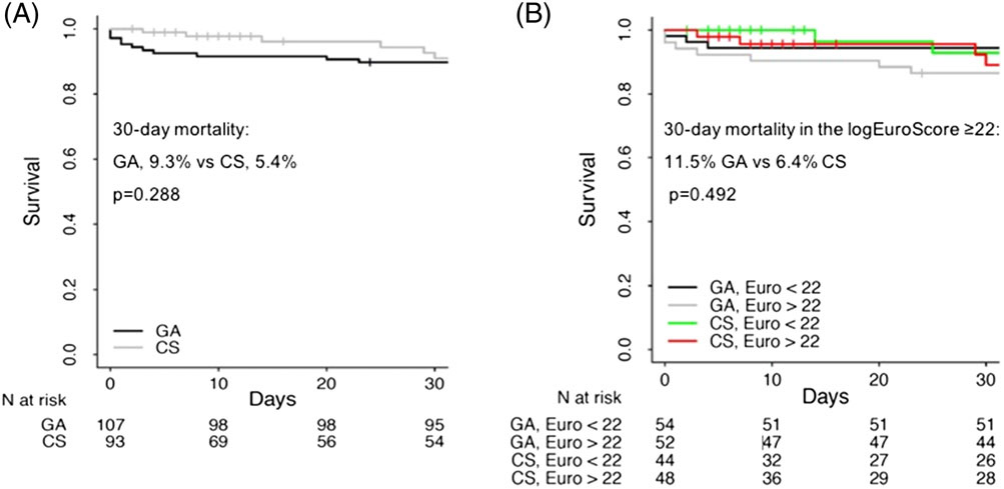
Survival curves of patients scheduled for TF‐TAVI. A, 30‐day survival in TF‐TAVI performed under general anaesthesia (GA) compared with conscious sedation (CS). B, 30‐day survival in TF‐TAVI performed under GA compared with CS in dependency of the logEuroSCORE I

**Figure 2:**
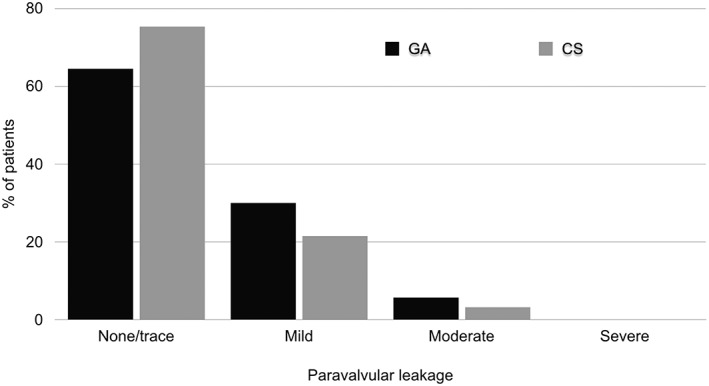
Severity of paravalvular leakage at post‐procedural day 7 in patients receiving general anaesthesia (GA) or conscious sedation (CS). Implanted type of valves in the GA‐group: Sapien XT, 91.5%; Sapien‐3, 6.5%; CoreValve, 2% and in the CS‐group: Sapien XT, 31.2%, Sapien‐3, 66.7%, CoreValve, 2.1%

All peri‐procedural complications, main peri‐procedural time courses, and mortality variables are shown in Table 3. No significant differences were seen between the GA‐group and the CS‐group, except for the need for cardiopulmonary resuscitation (CPR), which was significantly higher in the GA‐group (CPR, GA 10 [9.3%] vs CS 0 [0%], *P*‐value = 0.027, t‐test). The analysis of the main post‐procedural key time courses like LOS in the operating room (OR) and LOS in the ICU showed significantly shorter median time periods in the CS‐group (LOS in the OR, GA 130 [115‐150] min vs CS 120 [110‐132] min, *P*‐value = 0.015, Mann‐Whitney‐U‐test; LOS ICU, GA 2.1 (1.0–2.0) d vs CS 1.6 (1.0–1.5) d, *P*‐value = 0.002, Mann–Whitney‐U‐test). No significant difference was observed for LOS in the hospital between the groups (*P*‐value = 0.083, Mann‐Whitney‐U‐test). The echocardiographic evaluation of the degree of residual paravalvular leakage, according to the recommendations of the Valve Academic Research Consortium‐2^26^ at day seven post‐procedural or at discharge (when the stay in the hospital was shorter than 7 days), showed no differences between both groups (Figure 2). With respect to 30‐day mortality, no significant difference was seen between the groups (GA 9.3% vs CS 5.4%, *P*‐value = 0.288, t‐test) (Figure 1A).

**Table 3:** Peri‐procedural complications, main time courses, and mortality

	Overall (*n* = 200)	GA‐Group (*n* = 107)	CS‐Group (*n* = 93)	*P‐*Value
Stroke or TIA *n* (%) a	12 (6)	8 (7.5)	4 (4.3)	0.346
Delirium *n* (%) a	8 (4)	4 (3.7)	4 (4.3)	0.839
Acute kidney injury *n* (%) a	20 (10)	10 (9.3)	10 (10.8)	0.741
New pacemaker permanent *n* (%) a	22 (11)	15 (14)	7 (7.5)	0.143
New atrial fibrillation *n* (%) a	10 (5)	5 (4.7)	5 (5.4)	0.820
Vascular complications *n* (%) a	41 (20.5)	20 (18.7)	21 (22.6)	0.497
Pericardial effusion *n* (%) a	12 (6)	7 (6.5)	5 (5.4)	0.729
Pericardial tamponade *n* (%) a	4 (2)	4 (3.7)	0	0.060
Acute myocardial ischemia *n* (%) a	5 (2.5)	3 (2.8)	2 (2.2)	0.768
CPR *n* (%) a	10 (5)	10 (9.3)	0	0.027
Conversion to open surgery *n* (%) a	2 (1)	2 (1.9)	0	0.185
Inotropics post‐procedural *n* (%) a	8 (4)	6 (5.6)	2 (2.2)	0.213
Glomerular filtration rate (ml min^−1^) b	50 (38‐59)	47 (34‐63)	55 (39‐60)	0.011
Length of stay in the OR (min) b	122 (110‐140)	130 (115‐150)	120 (110‐132)	0.015
Length of stay on ICU (d) b	1.9 (1.0‐1.8)	2.1 (1.0‐2.0)	1.6 (1.0‐1.5)	0.002
Hospital length of stay (d) b	10 (7‐13)	10 (7‐13)	8 (7‐12)	0.083
30‐d mortality, *n* (%) a	15 (7.5)	10 (9.3)	5 (5.4)	0.288

a Numbers (% of total in group; *P*‐value t‐test).

b Median (interquartile range; *P*‐value Mann‐Whitney‐U‐test).

Abbreviations: CPR, cardiopulmonary resuscitation; CS, conscious sedation; GA, general anaesthesia; ICU, intensive care unit; OR, operating room; TIA, transient ischaemic attack.

### 3.5 Intra‐procedural anaesthesia characteristics in patients with a logEuroSCORE *I* ≥ 22

In high‐risk patients with a logEuroSCORE I ≥ 22 (*n* = 99), differences between GA and CS in intra‐procedural anaesthesia characteristics are shown in the Table 4. In contrast to the unselected population, no differences were seen between the groups regarding anaesthesia time and procedural time.

**Table 4:** Intra‐procedural anaesthesia characteristics in patients at higher risk, defined by a logEuroScore ≥22

	GA‐Group (*n* = 52)	CS‐Group (*n* = 47)	*P‐*Value
Anaesthesia time (min) a	120 (105‐140)	110 (100‐135)	0.077
Procedural time (min) a	55 (40‐79)	50 (35‐70)	0.157
Propofol total (mg/kg) a	3.4 (2.9‐4)	0.4 (0.3‐0.5)	<0.0001
Remifentanil total (μg/kg) a	14.7 (11.8‐17.2)	1.8 (1.4‐2.4)	<0.0001
Volume total (mL/kg) a	9 (6‐12.8)	7 (5‐9)	0.011
Norepinephrine total (μg/kg) a	*n* = 50 2 (1.2‐4)	*n* = 42 0.1 (0.08‐1.1)	<0.0001
Akrinor given *n* (%) b	15 (28.28)	0	<0.0001
Amiodarone given *n* (%) b	5 (9.6)	2 (4.3)	0.339
Enoximon given *n* (%) b	1 (1.9)	3 (6.4)	0.619
MAP max (mmHg) c	97 (17.1)	97.3 (12.6)	0.488
MAP min (mmHg) c	56.2 (18.9)	74.5 (12.9)	<0.0001
HR max (/min) c	74.8 (17.7)	75.9 (16.0)	0.692
HR min (/min) c	49.1 (17.3)	61.1 (14)	<0.0001
SpO_2_ min (%) a	94 (91‐97)	93 (91‐96)	0.147
Red cell unit given =1 *n* (%) _b_	1 (1.9)	2 (4.3)	0.017
Red cell unit given >1 *n* (%) b	8 (15.4)	1 (2.1)	0.017
Platelets given *n* (%) b	3 (5.8)	0	0.128
FFP given *n* (%) b	0	0	na

a Median (interquartile range; *P*‐value Mann‐Whitney‐U‐test).

b Numbers (% of total in group; *P*‐value t‐test).

c Mean (standard deviation; *P*‐value t‐test).

Abbreviations: CS, conscious sedation; FFP, fresh frozen plasma; GA, general anaesthesia; HR, heart rate; MAP, mean arterial pressure; NA, not assessed.

Total amount of propofol and remifentanil was significantly higher in the GA‐group (*n* = 52) compared with the CS‐group (*n* = 47) (*P*‐value <0.0001, Mann–Whitney‐U‐test). Significantly more median (IQR) i.v. fluids (9 (6‐12.8) ml/kg vs 7 (5‐9) ml/kg, *P*‐value = 0.011, Mann‐Whitney‐U‐test) and more median (IQR) amount of norepinephrine (2 (1.2‐4) μg/kg vs 0.1 (0.08‐1.1) μg/kg, *P*‐value <0.0001, Mann‐Whitney‐U‐test) were administered in the GA‐group. We observed a significantly lower mean (SD) value of MAP in the GA group compared with CS (MAP, GA: 56.2 (18.9) mmHg vs MAP, CS: 74.5 (12.9) mmHg, *P*‐value <0.0001, t‐test). Additionally, transfusion rate of red blood cells in the GA‐group was significantly higher compared with the CS‐group.

### 3.6 Peri‐procedural complications, main peri‐procedural time courses, and mortality in patients with a logEuroSCORE *I* ≥ 22

In patients with a logEuroSCORE I ≥ 22, differences between GA (*n* = 52) and CS (*n* = 47) in peri‐procedural complications, main time courses, and mortality are shown in Table 5. Regarding peri‐procedural complications, the only significant difference between the GA and CS‐group was the more frequent necessity of CPR in the GA‐group compared with the CS‐group (9.6% vs 0%, *P*‐value = 0.039, t‐test). LOS in the OR and in the hospital did not differ between groups (LOS OR *P*‐value = 0.209; LOS hospital *P*‐value = 0.936, Mann‐Whitney‐U‐test), however, patients in the GA‐group had a significantly longer median (IQR) stay in the ICU compared with the CS‐group (2.6 (1.3‐2.3) d vs 2.0 (1.1‐2.0) d, *P*‐value = 0.003, Mann‐Whitney‐U‐test). With respect to 30‐day mortality, no difference was observed between the groups (GA 11.5% vs CS 6.4%, *P*‐value = 0.492, t‐test; Figure 1B).

**Table 5:** Peri‐procedural complications, main time courses, and mortality in patients at higher risk, defined by a logEuroScore ≥22

	GA‐Group (*n* = 52)	CS‐Group (*n* = 47)	*P‐*Value
Stroke or TIA *n* (%) a	6 (11.5)	2 (4.3)	0.274
Delirium *n* (%) a	4 (7.7)	3 (6.4)	0.800
Acute kidney injury *n* (%) a	6 (11.5)	4 (8.5)	0.741
New pacemaker permanent *n* (%) a	6 (11.5)	2 (4.3)	0.282
New atrial fibrillation *n* (%) a	4 (7.7)	1 (2.1)	0.820
Vascular complications *n* (%) a	10 (19.2)	12 (25.5)	0.478
Pericardial effusion *n* (%) a	3 (5.8)	3 (6.4)	0.898
Pericardial tamponade *n* (%) a	2 (3.8)	0	0.287
Acute myocardial ischemia *n* (%) a	2 (3.8)	1 (2.1)	0.618
CPR *n* (%) a	5 (9.6)	0	0.039
Conversion to open surgery *n* (%) a	0	0	na
Inotropics post‐procedural *n* (%) a	3 (5.8)	2 (4.3)	0.731
Glomerular filtration rate (mL/min) b	42.5 (34.7‐50)	48.0 (35.5‐60)	0.193
Length of stay in the OR (min) b	132 (115‐150)	125 (110‐155)	0.209
ICU length of stay (d) b	2.6 (1.3‐2.3)	2.0 (1.1‐2.0)	0.003
Hospital length of stay (d) b	11.5 (9‐14.8)	9.0 (7‐12)	0.936
30‐d mortality *n* (%) a	6 (11.5)	3 (6.4)	0.492

a Numbers (% of total in group; *P*‐value t‐test).

b Median (interquartile range; *P*‐value Mann‐Whitney‐U‐test).

Abbreviations: CPR, cardiopulmonary resuscitation; CS, conscious sedation; GA, general anaesthesia; ICU, intensive care unit; OR, operating room; TIA, transient ischaemic attack.

### 3.7 Cox regression analysis

A cox regression analysis was utilized to identify a number of variables that were independently related to mortality. In order to assess whether there is any additional effect by type of anaesthesia we added this parameter later to the analysis. Beside CPR (HR 6.85, 95% CI 6.243‐19.25, *P*‐value <0.001, Wald‐test), stroke or TIA (HR 3.20, 95% CI 1.20‐8.52, *P*‐value = 0.020, Wald‐Test), STS score (HR 2.52, 95% CI 1.42–5.30, *P*‐value = 0.01, Wald‐test), and acute kidney injury (HR 2.91, 95% CI 1.65‐7.34‐6.39, *P*‐value = 0.02, Wald‐test), the type of anaesthesia had no significant impact upon the survival time after TF‐TAVI (HR 0.82 95% CI 0.36‐1.88, *P*‐value =0.65, Wald‐test) (Table 6).

**Table 6:** Results from Cox regression analysis presented as hazard ratios with 95% CI

	Hazard Ratios	95% CI	*P‐*Value^a^
STS score	2.52	1.20‐5.30	0.020
CPR	6.85	2.43‐19.25	<0.0001
Stroke or TIA	3.20	1.20‐8.52	0.020
Acute kidney injury	2.91	1.65‐7.34	0.020
Anaesthesia (CS)	0.820	0.36‐1.88	0.650

Abbreviations: CPR, cardiopulmonary resuscitation; CS, conscious sedation; STS, Society of Thoracic Surgeons; TIA, transient ischaemic attack.

a Wald‐test.

## 4 DISCUSSION

The main finding of our registry‐derived, single‐center, before‐and‐after study is that conscious sedation for the TF‐TAVI procedure is generally feasible, safe, and non‐inferior compared with general anaesthesia with respect to peri‐procedural complications and 30‐day mortality.

CS, in contrast to GA, is characterized by a significantly higher degree of intra‐procedural balanced haemodynamics, mirrored by less i.v. fluids and catecholamines administered, and less red blood cells transfused. Moreover, the CS‐group stayed significantly shorter in the ICU, which has been shown to be an independent risk factor of death in the first 30‐days after the procedure.^27^

Worldwide, approximately 90% of the TF‐TAVIs are performed under GA due to several reasons. One of them might be the safe and reliable working conditions for the cardiologist, especially due to the avoidance of sudden, uncontrolled movements of the patient under CS that might endanger the success of the procedure. The frequent concerns expressed about the safety of CS‐concepts for TF‐TAVI, especially in light of acute procedural‐related complications that need immediate modification of the anaesthesia management, have recently been refuted by several investigations, and similarly highlighted by our results.^10^, ^14^, ^28^, ^29^ With respect to the necessity to convert from CS to GA, we report on a conversion of 5 times (5.1%). In 4 cases (4.3%), conversion from CS to GA was related to procedural complications, and in only 1 patient (1.1%) due to persistent agitation. The low conversion rate of 1.1% specifically related to this CS‐concept underlines its safety and effectiveness. These data are in good agreement with Yamamoto et al showing a 4.6% conversion rate due to procedure‐related complications and no conversion based on the non‐effectiveness of the sedation‐concept.^28^ It has been shown that the feasibility and safety of different CS‐concepts are at least non‐inferior compared with GA‐concepts regarding peri‐procedural complications and 30‐day mortality. In this respect, our results are in agreement with previous investigations, showing no differences between the two discussed anaesthesia techniques.^10^, ^14^ Another important point in favour of GA, might be the use of TOE guidance throughout the procedure, especially after the implantation, to grade residual paravalvular regurgitation and for potential management of complications.^30^ However, none of the available research on this subject has provided evidence that the procedural success, with respect to positioning of the valve and the frequency of relevant paravalvular regurgitation, is higher in patients under CS without the use of TOE. Although this is not the main focus of the present manuscript, we can report that we saw no differences between the groups regarding success of positioning of the valve or a higher percentage of misinterpretation of valve regurgitation in the CS‐group (Figure 2).

Another interesting observation in the context of CS, beyond the improved haemodynamics ‐evidenced by lower requirements of catecholamines during the procedure‐, is the significantly reduced amount of total i.v. fluids needed to achieve this stability, together with a reduced amount of red blood cell transfusion. These findings have also been described previously.^15^ In addition, Goren et al found significantly less episodes of sepsis in the CS‐group,^10^ possibly related to significantly less red blood cell transfusion, a supposable relationship that has been described previously in cardiac and non‐cardiac surgery patients.^31^, ^32^ Secondly, they found a trend towards reduced post‐procedural pulmonary complications, likewise to be related to red blood cell transfusion.^31^ However, the pre‐procedural median (IQR) haemoglobin (Hb) values (GA, Hb 12.3 [10.9‐13.1] mg/dL; CS, Hb 12.3 [10.8‐13.3] mg/dL) and post‐procedural median (IQR) Hb‐values (GA, Hb 10.3 [9.2‐11.3] mg/dL; CS, Hb 10.4 [9.4‐11.7] mg/dL) did not differ. Another supporting point could be the more restrictive intra‐procedural fluid management in the CS‐group; this has been reported in other investigations.^10^, ^15^

In a recent study by Mayr et al, the authors observed adverse events in the CS‐group, like bradypnoea (52%) and the need for airway protection manoeuvres (36%) or bag‐mask‐ventilation (19%), more often than in the GA group.^33^ These findings are in contrast to previously reported data, our study included.^10^, ^14^, ^29^ Analyzing the different amounts of remifentanil administered in the CS‐group of both studies (remifentanil total, Mayr et al: 4.5 [3.7‐5.2] μg/kg; our study: 1.8 [1.4‐2.5] μg/kg), might help to explain the higher tendency towards respiratory complications in the Mayr‐study, leading to a significant longer stay in the ICU compared with the majority of previously reported data. In contrast, Bergmann et al reported significantly less requirement of postoperative mechanical ventilation in the CS‐group.^14^ These findings gave an idea of substantially different CS‐concepts regarding choice of drug and dosage compared with the more homogenous way to perform GA.

To the best of our knowledge, this study is the first to compare the effects of GA or CS in TF‐TAVI patients at higher surgical risk, defined by a logEuroSCORE *I* ≥ 22% (median), with respect to peri‐procedural outcome. Even in this patient population, we have observed similar results in favor of the CS‐group. However, among the key time courses, only LOS in the ICU was significantly shorter. No differences were seen in the post‐procedural complication rate and in 30‐day mortality. Especially, the aspect that patients need to stay significantly less time on intensive care following CS have to be interpreted in consideration of the results of the multivariate Cox regression analysis, yielding a number of factors that were associated with an increased risk of peri‐procedural death. Other associated factors beyond LOS in the ICU were the need of CPR, stroke or TIA, STS score, and acute kidney injury. The type of anaesthesia, GA or CS, appears to have no significant impact upon the survival time after TF‐TAVI.

With the present study, we were able to show that the implementation of a CS‐concept could lead to significantly reduced key time periods like anaesthetic time, and LOS in the OR and in the ICU. Although not the primary aim of our investigation, reduced time courses have been shown to effectively reduce costs.^34^ Although there might be some high level contemporary departments where the LOS in hospital for patients after TF‐TAVI is somewhere between 1 and 3 days^35^, most of the available literature reports LOS in hospital between 7 and 13 days, which is in agreement with our data.^36^, ^37^, ^38^

The present study has several limitations. This is a single‐center observation in a before‐after‐design with a limited number of patients. Due to the before‐after‐design, the two discussed anaesthesia regimes were not randomized, but the result of the departmental decision to change anaesthesia management for TF‐TAVI from GA to CS at a given time. This was done in order to further develop our anaesthesia concept, helping to improve patient safety. Another point of criticism relates to the ongoing “learning curve” of the cardiologist, on the one hand, and the ongoing “learning curve” of the anaesthesiologist responsible for the CS‐concept, on the other. The finding that every tenth patient in the GA‐group was in need of CPR might also be interpreted as an indicator of the ongoing “learning curve.” However, continuous improvement of manual, technical, and logistic skills are basic requirements for enhanced patients' safety and, consequently, can never be ruled out. Since there is only one randomized controlled trial available for the moment on this issue,^33^ the abovementioned limitations similarly apply to most of the studies in the recent past. In addition, the device used has changed in February 2014 from the Edwards Sapien XT to the Sapien‐3. Among other changes, the new device features a lower profile delivery system and a paravalvular sealing system. The latter factors might, thus, influence the rate of vascular and bleeding complications, new pacemaker implants, and the occurrence of paravalvular leaks between the two groups.

## 5 CONCLUSION

In summary, this study provided evidence that CS in patients scheduled for TF‐TAVI is feasible, safe, and non‐inferior regarding peri‐procedural complications and post‐procedural 30‐day mortality compared with GA, even in patients at higher surgical risk. Moreover, implementing this CS‐concept led to significantly reduced key time periods like anaesthesia time, LOS in the OR, and LOS in the ICU.

## FUNDINGS

The present study was supported by the Department of Cardiology and Angiology, University Hospital Schleswig‐Holstein, Campus Kiel and by the Department of Anaesthesiology and Intensive Care Medicine, University Hospital Schleswig‐Holstein, Campus Kiel, Germany.

## CONFLICTS OF INTEREST

None.

## AUTHOR CONTRIBUTIONS

Conceptualization: Derk Frank, Jochen Renner

Formal analysis: Anna Tesdorpf, Sandra‐Freitag‐Wolf, Derk Frank, Jochen Renner, Helga Francksen, Rainer Petzina, Georg Lutter, Norbert Frey

Writing – original draft preparation: Jochen Renner

Writing – review and editing: Derk Frank, Jochen Renner, Georg Lutter, Rainer Petzina, Norbert Frey, Helga Francksen, Anna Tesdorpf, Sandra Freitag‐Wolf

## Supporting information


**Table S1:**
Patients in which conversion from conscious sedation to general anaesthesia was needed.
