# Supplementary material for: A retrospective study of conscious sedation versus general anaesthesia in patients scheduled for transfemoral aortic valve implantation: A single center experience
Source: Health Sci Rep. 2018 Nov 1;2(1):e95. doi: 10.1002/hsr2.95 (PMC6346987; doi:10.1002/hsr2.95)
Supplement: Supplementary file 1 — Table S1: Patients in which conversion from conscious sedation to general anaesthesia was needed. [file HSR2-2-e95-s001.docx]

| **Supplementary Table S1:** Patients in which conversion from conscious sedation to general anaesthesia was needed. | | | | | |
| --- | --- | --- | --- | --- | --- |
|  | Patient 1 | Patient 2 | Patient 3 | Patient 4 | Patient 5 |
| Age (yr) | 86 | 76 | 91 | 77 | 88 |
| BMI (kg/m^2^) | 20 | 28 | 24 | 28 | 26 |
| Sex | female | male | female | female | female |
| logEuroSCORE I (%) | 70 | 19 | 43 | 12 | 12 |
| STS score (%) | 6 | 4 | 14 | 2 | 6 |
| Reason for conversion | persistent hypotonia post implantation CPR | persistent agitation | myocardial ischemia | myocardial ischemia | ventricular perforation |
| Survived/died | died | survived | died | survived | died |

BMI, body mass index; EuroScore, European System for Cardiac Operative Risk Evaluation; STS score, Society of Thoracic Surgeons.
